# Efficacy and Safety of Umifenovir (Arbidol) in Children with Influenza-like Illnesses: A Systematic Review and Meta-Analysis

**DOI:** 10.3390/pediatric18030077

**Published:** 2026-06-09

**Authors:** Vilya Bulgakova, Artem Poromov, Irina Leneva, Natalia Pshenichnaya

**Affiliations:** 1 Institute of Motherhood and Childhood, Pirogov Russian National Research Medical University, 117513 Moscow, Russia; 2 Pediatrics and Child Health Research Institute, Petrovsky National Research Center of Surgery, 119991 Moscow, Russia; 3 Virology Department, Mechnikov Research Institute of Vaccines and Sera, 105064 Moscow, Russia; 4 Medical Institute, Peoples’ Friendship University of Russia Named After Patrice Lumumba, 117198 Moscow, Russia; 5 Department of Infectious Diseases, Russian Medical Academy of Continuing Professional Education, Federation, 125993 Moscow, Russia; 6 Central Research Institute of Epidemiology, Federal Service for Surveillance on Consumer Rights Protection and Human Wellbeing (Rospotrebnadzor), 111123 Moscow, Russia; 7 Moscow Regional Clinical Researched Institute Named After M.F. Vladimirsky, 129110 Moscow, Russia

**Keywords:** umifenovir, Arbidol, influenza-like illness, children, systematic review and meta-analysis

## Abstract

**Background:** Pediatric influenza-like illness (ILI) represents a major global health burden. However, international treatment guidelines lack robust evidence specific to children. Umifenovir (Arbidol) is a broad-spectrum antiviral approved for pediatric use in several countries, but clinical data are fragmented and regionally limited. **Methods:** A comprehensive search of PubMed, Russian (RSCI, national archives, regulatory documents), and Chinese (CNKI) databases was conducted for pediatric randomized controlled trials (RCTs) and non-randomized trials comparing umifenovir to symptomatic therapy (ST) or oseltamivir. Risk of bias was assessed using the RoB 2 tool for RCTs, and ROBINS-I for non-RCTs. Outcomes included the duration of fever and other symptoms, prophylactic efficacy, and adverse events. Random-effects models were used (Hartung–Knapp–Sidik–Jonkman approach). The review was not registered. **Results:** We included 16 therapeutic and eight prophylactic trials enrolling approximately 4700 and 2000 children, respectively. Compared with ST, umifenovir reduced the duration of fever (MD −1.41 days, 95% CI: −1.78 to −1.05), cough (−1.15 days, 95% CI: −1.50 to −0.79), and hospitalization. The complication risk decreased (RR 0.34, 95% CI: 0.23–0.51). For prophylaxis, umifenovir reduced the risk of ILI (RR 0.68, 95% CI: 0.54–0.87) and laboratory-confirmed influenza (RR 0.41, 95% CI: 0.29–0.59). Adverse events were generally mild and did not differ significantly from ST or oseltamivir (RR 0.78, 95% CI: 0.51–1.20). **Conclusions:** Umifenovir may reduce symptom duration, complications, and infection risk in pediatric ILI, with a favorable safety profile. However, the overall certainty of evidence is limited by the age of the studies, geographic restriction, and methodological quality.

## 1. Introduction

Influenza and influenza-like illness (ILI) remain major contributors to morbidity among children worldwide. ILI in pediatric populations is clinically defined as acute onset of fever accompanied by respiratory symptoms such as cough, sore throat, or rhinorrhea, and may be caused not only by influenza viruses but also by respiratory syncytial virus, metapneumovirus, rhinoviruses, coronaviruses, and adenoviruses [1]. Each year up to one-third of school-aged children develop ILI. For example, the incidence of acute upper respiratory tract infections among children aged 0–14 years reaches 75–81 thousand per 100,000 in Russia [2]. Early differentiation of influenza from other viral etiologies in children is challenging due to overlapping clinical features, and although PCR-based testing provides high accuracy, it is not always available for routine pediatric care. The high burden of ILI in children and the limitations of diagnostic approaches underscore the ongoing need for effective antiviral and preventive strategies [3].

At present, no globally recognized recommendations for pediatric antiviral treatment exist. Although the World Health Organization (WHO) updated its Clinical Practice Guidelines for Influenza in 2024 [4], the recommendations remain largely based on evidence from adult populations and do not include a dedicated evaluation of antiviral therapies for children. Compared to the 2022 version [5], the updated guidelines mention, for the first time, baloxavir, peramivir, umifenovir and others. The WHO guidelines also note that “insufficient human data are available for the use of umifenovir in children,” underscoring the absence of a pediatric-specific evidence base.

Umifenovir is a broad-spectrum antiviral agent developed in Russia in the 1980s. It has demonstrated in vitro and in vivo activity against a wide range of viruses, including influenza A and B [6,7], coronaviruses [8], respiratory syncytial virus, adenovirus, coxsackievirus [9], and some others. During the COVID-19 pandemic, interest in umifenovir increased due to reports of potential activity against SARS-CoV-2 [10].

Original umifenovir (Arbidol) was approved for the treatment and prevention of influenza and acute respiratory viral infections (ARVIs) in adults in 1988 and in children in 1996 [11]. Umifenovir is also registered in other Eurasian Economic Union countries (Kazakhstan, Belarus, Armenia, and Kyrgyzstan); in Uzbekistan, based on the Russian registration dossier and approval for use from two years old (form for suspension preparation); and in China, based on its own preclinical and clinical studies [12], including several studies involving children [13,14]. The single dose of the drug, depending on age, was as follows: 50 mg for children aged 3–6 years, 100 mg for children aged 6–12 years, and 200 mg for adolescents over 12 years, administered four times daily for the treatment of influenza and other ILI. Umifenovir is not approved in the European Union or the United States, and its international regulatory adoption remains limited.

Adult prophylactic and therapeutic clinical trials (CTs) for the treatment of influenza and other acute respiratory infections (ARIs) have included several randomized, placebo-controlled studies [15,16], some of which were conducted outside Russia [17,18,19]. Although umifenovir has been used in both adults and children, evidence specific to pediatric populations remains heterogeneous and fragmented, supporting the need for a dedicated systematic review.

Pediatric clinical trials conducted at multiple centers, including the Institute of Virology (Moscow) and the Influenza Institute (Saint Petersburg), spanned three epidemic seasons (1993–1995) [20]. These and others CT results were published in Russian-language journals, limiting their visibility to the international scientific community, as many of these are not indexed in international databases and remain inaccessible to the global scientific community. To address this gap, we conducted a comprehensive, historically inclusive literature search, incorporating not only international databases but also Russian national sources such as the Russian Science Citation Index (RSCI), archives of domestic medical journals, and original clinical trial reports. China represents the second major source of clinical evidence on umifenovir, including pediatric studies, making Chinese databases essential for capturing the full scope of available data.

Given the widespread use of umifenovir in children in Russia and China, the heterogeneous and regionally fragmented evidence base, and continuing uncertainty reflected in recent WHO guidelines, a comprehensive synthesis of pediatric data is needed. The aim of this systematic review and meta-analysis is to evaluate the efficacy and safety of umifenovir for the treatment and prevention of influenza-like illnesses (ILIs) and influenza in children.

## 2. Materials and Methods

### 2.1. Search Strategy

This systematic review and meta-analysis was conducted and reported in accordance with the PRISMA 2020 statement. The PRISMA 2020 for Abstracts Checklist is provided in the Supplementary Materials (Table S1). The review protocol was not prospectively registered. However, the study methodology, eligibility criteria, outcomes, and analytical approach were predefined before data extraction and analysis. The lack of prospective protocol registration should nevertheless be considered a limitation of this review. The search strategies for all databases are provided below.

PubMed, Russian Science Citation Index (RSCI, www.elibrary.ru, accessed on 10 July 2025), and the China National Knowledge Infrastructure (CNKI, oversea.cnki.net, accessed on 5 October 2025.) databases were systematically searched.

Search terms included English and Russian keywords for PubMed and RSCI related to the intervention and target conditions. The terms used were: “Arbidol”, “umifenovir”, “respiratory viral infection”, “influenza”, “ARVI”, “antiviral therapy”, “children” and related synonyms. Boolean operators (AND, OR) were applied to combine terms appropriately. Keywords used for CNKI were: “Arbidol”, as well as its Chinese-language equivalents: “阿比朵尔” (Abidol) and “盐酸阿比多尔/盐酸阿比朵尔” (Arbidol hydrochloride). Chinese-language studies identified through CNKI were screened by a native Chinese-speaking expert and translated into Russian for data extraction. The accuracy of extracted data and study interpretation in the final dataset and English-language manuscript were also verified by a native Chinese speaker. Reference lists of all included articles and relevant reviews were screened manually to identify any additional eligible studies not captured in the initial search. There were two investigators who independently screened and reviewed each study (A.P. and I.L.).

For studies identified through the RSCI, full-text electronic versions were manually reviewed where paper copies were not available. In addition, manual searches of archives of major Russian-language medical journals published between 1990 and 2000 were conducted to identify potentially relevant studies that may not have been indexed in the electronic RSCI database (July–August 2025).

The results of the registration studies were summarized in the monograph by Guskova et al. (1999) [20]. Original trial reports (regulatory documentation related to the original registration of umifenovir (Arbidol) in the Russian Federation) were additionally reviewed to obtain detailed information on study design, patient groups, and outcome assessment, as well as to verify the reported results. These materials included full clinical trial reports and supporting clinical data submitted for regulatory approval in children. Importantly, the development of umifenovir and the corresponding clinical studies were conducted within the framework of state-funded research programs assigned to public scientific institutions. Trial reports were submitted directly to the Ministry of Health of the USSR through the Pharmaceutical Committee as part of the mandatory regulatory process. Archival searches were performed based on references contained in the historical registration dossier, allowing identification of original clinical trial reports preserved in institutional and governmental archives.

### 2.2. Inclusion and Exclusion Criteria

Studies were considered eligible for inclusion if they met the following criteria: focused on children; evaluated the therapeutic effect of umifenovir in laboratory-confirmed influenza or clinically diagnosed ILI (fever with respiratory symptoms); randomized controlled trial (RCT) design or a comparative observational study (prospective or retrospective cohort, case–control, or cross-sectional); reported at least one quantifiable clinical or virologic outcome related to efficacy or safety.

Studies were excluded if they met any of the following criteria: preclinical investigations (in vitro or animal studies); publications focused on the pharmacological mechanism or pharmacokinetic–pharmacodynamic assessments; or other non-clinical formats (e.g., editorials, reviews, letters to the editor).

### 2.3. Study Selection and Data Extraction

Two reviewers (A.P. and I.L.) independently screened the titles and abstracts retrieved from the search, followed by full-text screening of potentially relevant articles. Discrepancies were resolved through discussion or consultation with a third investigator.

For each included study, the following data were extracted using a standardized data collection form:

- First author and year of publication;
- Country of study and study design;
- Patient characteristics (sample size, age, diagnosis);
- Control group intervention (placebo, standard of care or oseltamivir);
- Outcomes measured and effect sizes reported.

### 2.4. Outcomes

The outcomes were selected to evaluate the therapeutic and prophylactic effects of umifenovir in children with influenza-like illness (ILI). Primary efficacy outcomes included:

- Duration of fever (defined as the time from onset of fever (>37.5 °C) to sustained body temperature < 37.5 °C for at least 24 h, days);
- Cough duration, defined as time (days) to complete dry cough.

Secondary outcomes included the duration of symptom groups:

- Intoxication symptoms, defined as time (days) to complete resolution for all of these symptoms: fatigue, weakness, reduced appetite, headache, myalgia, and chills.
- Nasopharyngeal and catarrhal symptoms, defined as time (days) to complete resolution for all upper respiratory tract manifestations, including nasal breathing, rhinitis, dry mucous membranes, sore throat, scratchy or dry cough.
- Total illness duration (time to complete resolution of clinical symptom, days). Complete symptom resolution was defined as the absence of all predefined ILI-related symptoms as assessed by investigators using standardized clinical assessment forms.

Additionally, the proportion of patients achieving complete symptom resolution within a defined period and the time to symptom relief were key clinical endpoints. Virologic outcomes included the PCR negativity rate at specific time points (e.g., Days 4–5), serving as a marker of viral clearance.

Several studies reported complications and disease progression, including the incidence of pneumonia (bacterial or post-viral), bronchitis, sinusitis, or otitis. Length of hospital stay was assessed where data were available.

Adverse events (AEs) were collected in all trials reporting safety outcomes and included both general AE incidence and serious adverse events when specified.

Furthermore, several included studies evaluated the prophylactic use of umifenovir, with outcomes such as the incidence of laboratory-confirmed influenza or ILI incidents.

### 2.5. Risk of Bias Assessment

To evaluate the methodological quality of randomized controlled trials (RCTs) included in this analysis, we employed the revised Cochrane Risk of Bias tool (RoB 2) for randomized trials [21]. The risk of bias of the included non-randomized studies was assessed using the ROBINS-I tool [22].

Formal statistical tests for publication bias (e.g., Egger’s test) were not performed because all meta-analyses included fewer than 10 studies and exhibited substantial heterogeneity, conditions under which such tests are considered unreliable.

### 2.6. Statistical Analysis

All statistical analyses were performed using MetaAnalysisOnline (A5 Genetics Ltd., Hungary) an online tool for the rapid meta-analysis of clinical and epidemiological studies [23]. The random-effects model was used. Only primary analysis outcomes from randomized controlled trials (RCTs) were tested. Non-randomized controlled trials (non-RCTs) were synthesized separately and then combined in exploratory analyses with RCTs using subgrouped meta-analysis (subgroup = study design). Between-study variance was estimated using a Sidik–Jonkman estimator and 95% CIs were calculated with the Hartung–Knapp method. Heterogeneity was quantified with I^2^ and prediction intervals were reported.

Sensitivity analyses excluded studies at critical/high risk of bias and excluded unadjusted non-RCT estimates. For continuous outcomes measured on comparable scales, mean differences (MDs) with 95% confidence intervals (CIs) were calculated, as this approach provides more interpretable and robust estimates than standardized mean differences when outcomes can be expressed on a common scale. For dichotomous outcomes, risk ratios (RRs) with 95% CIs were computed. All analyses reported two-sided *p*-values with α = 0.05.

## 3. Results

### 3.1. The Characteristics of Studies

**Figure 1:**
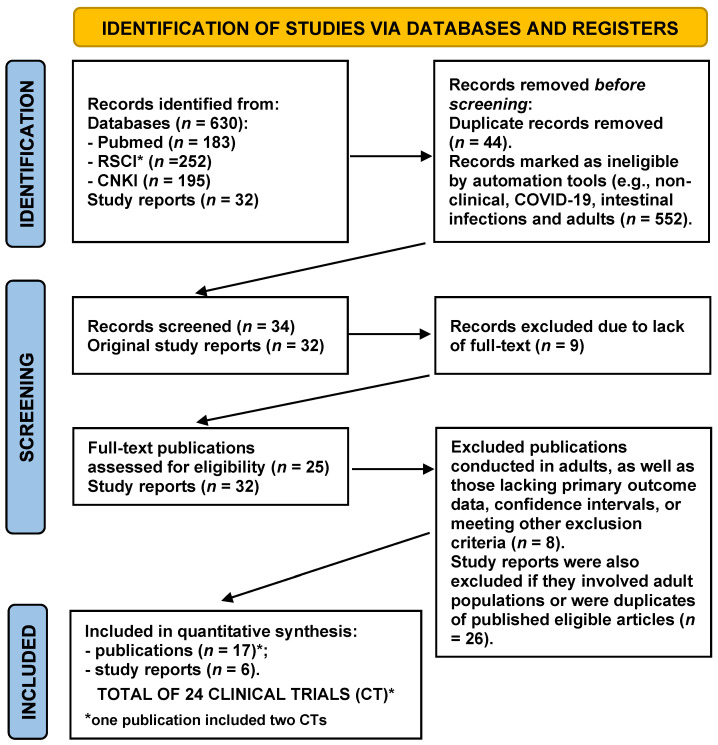
Study flow diagram. Original study reports were provided for analysis by the marketing authorization holder of the original umifenovir product (Arbidol) in the Russian Federation (* including archives of major Russian medical journals).

Figure 1 shows the literature search flow, removal of duplicates, and screening based on title, abstract, full text, and original study reports provided by the manufacturer of the original umifenovir drug Arbidol. As a result, 24 clinical trials (CTs) assessing the efficacy and safety of umifenovir in the treatment and prevention of influenza and influenza-like illnesses in children were included. These studies represent a range of randomized controlled trials (RCTs), prospective and retrospective (non-RTC) studies conducted between 1993 and 2024 in Russia, Ukraine, and China. In total, approximately 4700 participants were enrolled in the therapeutic studies and nearly 2000 in the prophylactic studies.

Among the 16 therapeutic CTs (Table A1), at least seven were randomized controlled trials, including both symptomatic treatment and active comparator (oseltamivir) designs. Additional evidence was provided by prospective and retrospective cohort studies. Study populations varied by setting, with both hospitalized and outpatient children represented. Most therapeutic studies focused on patients with laboratory-confirmed influenza or clinically diagnosed ARVIs.

The prophylactic dataset included eight studies (Table A1). Most were single-center trials conducted during seasonal influenza and ARVI outbreaks in Russia, with the exception of one Ukrainian study [24]. Study populations typically comprised healthy children in schools or organized groups. The results of the risk of bias assessment using the Cochrane Collaboration tool are presented in Table A1 and Table A2.

The approved umifenovir regimen for influenza and influenza-like illness (ILI) is age-specific single doses (50 mg for children aged 3–6 years, 100 mg for 6–12 years, and 200 mg for >12 years) administered four times daily for 5 days in Russia and three times daily for 5 days in China. In prophylactic studies, umifenovir was administered one to three times weekly for 3–5 weeks (Table A1 and Table A2).

### 3.2. Outcomes

The outcomes extracted from the included studies encompassed a broad range of clinical, virological, and safety measures relevant to both therapeutic and prophylactic evaluation of umifenovir. The most frequently reported clinical endpoints were fever duration, intoxication duration, and catarrhal symptom duration, each assessed in multiple independent trials. Additional commonly evaluated outcomes included cough duration, overall complication rate, and the incidence of bacterial complications such as pneumonia and bronchitis.

In prophylactic studies, the primary endpoints were the incidence of influenza and ARVI, including recurrent infections, often measured during epidemic or seasonal outbreaks. Several trials also incorporated virological outcomes, such as viral antigen detection and PCR negativity rates, providing objective evidence of antiviral activity.

Safety was consistently addressed across therapeutic trials, with adverse events systematically reported in randomized controlled and observational studies.

### 3.3. Clinical Symptoms

All patients had fever. In hospitalized studies, children were generally admitted within the first 2 days after onset of influenza-like illness (ILI) symptoms. Clinical manifestations were characterized by intoxication syndrome and respiratory tract involvement, including hyperemia of the oropharyngeal mucosa, and painful nonproductive cough in most patients. Children were assessed using standard clinical approaches for influenza and ILI. Clinical status and symptom dynamics were monitored daily throughout the treatment period using predefined clinical assessment criteria.

#### 3.3.1. Fever Duration

**Figure 2:**
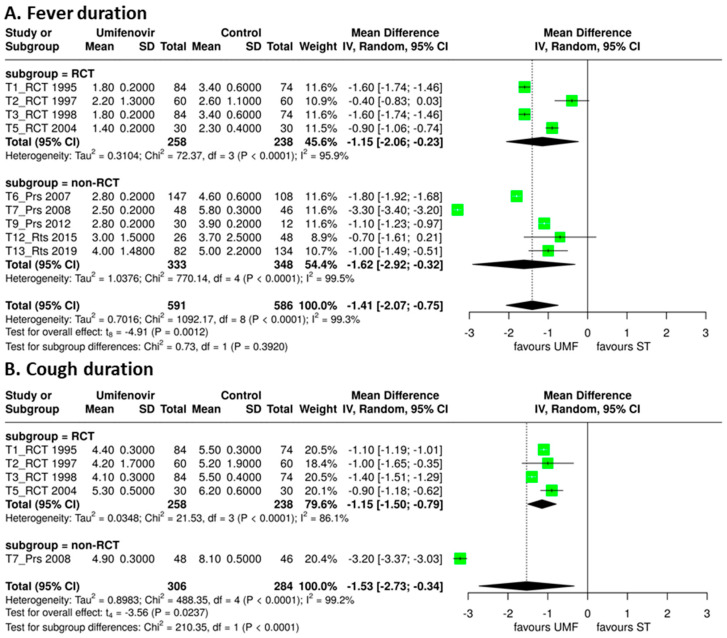
Forest plot comparing the duration of fever (**A**), cough (**B**). UMF—umifenovir, ST—symptomatic therapy. Random-effects model and Sidik–Jonkman heterogeneity used estimator with Hartung–Knapp adjustment. Study identifiers correspond to those listed in Appendix A, Table A1.

Nine studies including 591 subjects in the UMF cohort and 586 in the ST cohort evaluated fever duration (Figure 2A). The pooled analysis using a random-effects model with the inverse variance method demonstrated a significant reduction with UMF compared to ST (a mean difference (MD) −1.41 days; 95% CI: −1.97 to −0.84; *p* < 0.05). The summarized raw mean (MRAW) was 2.46 days (95% CI: 2.06–2.85) for UMF and 3.86 days (95% CI: 3.01–4.70) for ST. Substantial heterogeneity was observed (χ^2^ *p* < 0.01; I^2^ = 99%), indicating high inconsistency in effect size estimates across studies. The funnel plot does not indicate a potential publication bias.

In the subgroup analysis by study design, RCTs demonstrated a mean reduction in fever duration of −1.09 days (95% CI: −1.49 to −0.68), while non-RCTs showed a somewhat greater effect of −2.07 days (95% CI: −3.34 to −0.80). However, the test for subgroup differences was not statistically significant (χ^2^ = 2.07, df = 1, *p* = 0.15), indicating no evidence of systematic variation between RCTs and observational studies.

#### 3.3.2. Cough Duration

Five studies including 306 subjects in the UMF cohort and 284 in the ST cohort assessed cough duration (Figure 2B); all children were hospitalized with ILI. The pooled analysis using a random-effects model with the inverse variance method showed a significant reduction with UMF compared to ST (MD −1.53 days; 95% CI: −2.38 to −0.68; *p* < 0.05). The summarized MRAW was 4.59 days (95% CI: 4.21–4.97) for UMF and 6.11 days (95% CI: 5.17–7.05) for ST. Substantial heterogeneity was detected (χ^2^ *p* < 0.01; I^2^ = 99%), indicating marked inconsistency in effect size estimates across studies. In subgroup analyses stratified by study design, both randomized controlled trials and non-randomized studies showed reductions in outcome duration; however, the effect estimate was larger in non-RCTs (MD −1.53 [−2.73 to −0.34]) than in RCTs (MD −1.15 [−1.50 to −0.79]), with a statistically significant difference between subgroups (test for subgroup differences: χ^2^ = 210.35, df = 1; *p* < 0.0001).

#### 3.3.3. Intoxication Duration

Five studies including 427 subjects in the UMF cohort and 404 in the ST cohort assessed intoxication duration. The pooled analysis using a random-effects model with the inverse variance method showed a significant reduction with UMF compared to ST (MD −1.37 days; 95% CI: −1.83 to −0.92; *p* < 0.05). The summarized MRAW was 2.77 days (95% CI: 2.14–3.40) for UMF and 4.12 days (95% CI: 3.66–4.58) for ST. Substantial heterogeneity was detected (χ^2^ *p* < 0.01; I^2^ = 99%), indicating high variability in effect size estimates across studies.

#### 3.3.4. Nasopharyngeal and Catarrhal Symptom Duration

Five studies including 427 subjects in the UMF cohort and 404 in the ST cohort assessed the duration of nasopharyngeal and catarrhal symptoms. The pooled analysis using a random-effects model with the inverse variance method demonstrated a significant reduction with UMF compared to ST (MD −1.10 days; 95% CI: −1.48 to −0.72; *p* < 0.05). The summarized MRAW was 5.45 days (95% CI: 4.34–6.56) for UMF and 6.63 days (95% CI: 5.72–7.55) for ST. Substantial heterogeneity was detected (χ^2^ *p* < 0.01; I^2^ = 95%), indicating inconsistency in effect size estimates across studies.

#### 3.3.5. Hospitalization Duration

Three studies including 142 subjects in the UMF cohort and 115 in the ST cohort assessed hospitalization duration. The pooled analysis using a random-effects model with the inverse variance method showed a significant reduction with UMF compared to ST (MD −0.91 days; 95% CI: −1.44 to −0.37; *p* < 0.05). The summarized MRAW was 7.17 days (95% CI: 4.56–9.77) for UMF and 8.10 days (95% CI: 6.09–10.11) for ST. Substantial heterogeneity was detected (χ^2^ *p* < 0.01; I^2^ = 84%), indicating variability in effect size estimates across studies.

### 3.4. Complication

Eight studies evaluated complication rates. UMF was associated with 109 events over 1312 persons, yielding a pooled incidence rate of 0.11 (95% CI: 0.06–0.16). In contrast, ST was associated with 196 events over 489 persons, corresponding to a pooled incidence rate of 0.38 (95% CI: 0.23–0.53). Both analyses demonstrated significant heterogeneity (UMF: I^2^ = 79%; ST: I^2^ = 89%), suggesting that variability among studies arose largely from differences in magnitude and/or direction of effect rather than random error.

**Figure 3:**
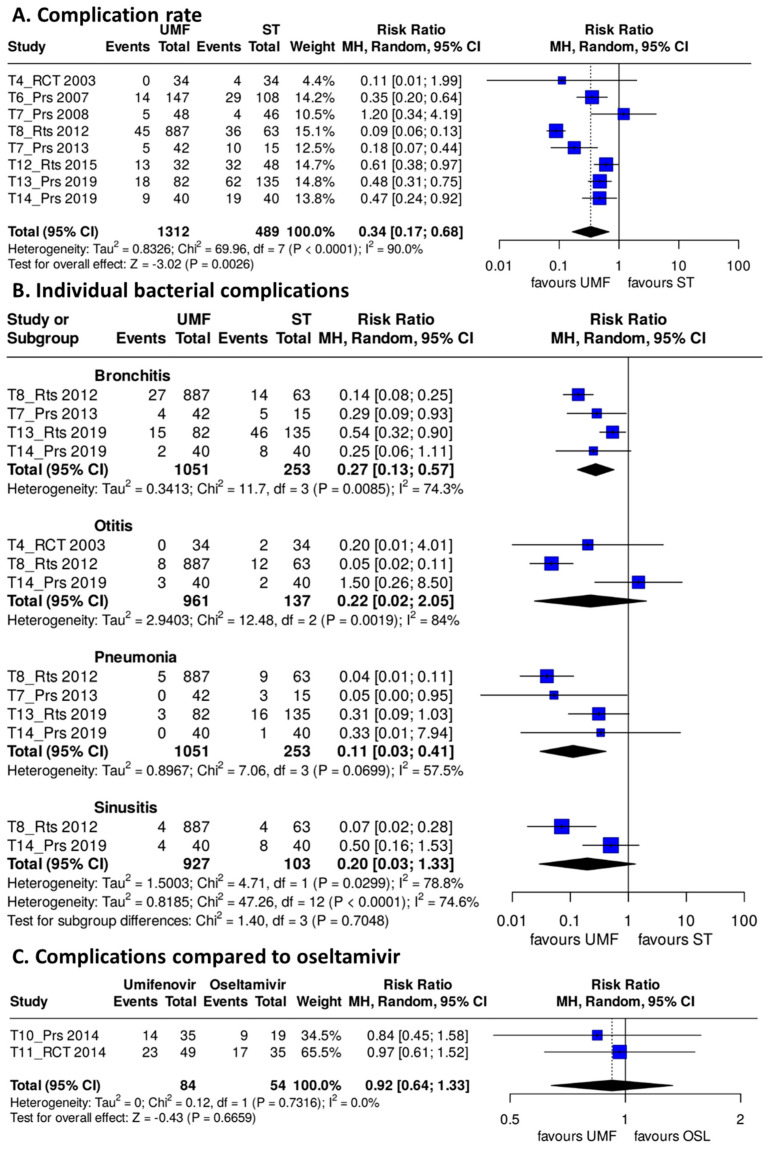
Forest plots of complication outcomes in patients receiving umifenovir (UMF) compared with standard therapy (ST) or oseltamivir. (**A**) Incidence of all reported complications. (**B**) Incidence of bronchitis, pneumonia, otitis, and sinusitis. (**C**) Comparison of overall complication rates between UMF and oseltamivir. Study identifiers correspond to those listed in Appendix A, Table A1.

The pooled analysis using a random-effects model with the Mantel–Haenszel method demonstrated that UMF significantly reduced the risk of complications compared with ST (RR: 0.34; 95% CI: 0.17–0.68; *p* < 0.05, Figure 3A). Substantial heterogeneity was observed (χ^2^ *p* < 0.01; I^2^ = 90%), indicating inconsistency in effect size estimates.

Umifenovir significantly reduced the risk of bronchitis compared with ST (RR = 0.27, 95% CI: 0.13–0.57; *p* < 0.01), with incidence rates of 4.6% (95% CI: 3.4–6.1%) in the UMF group versus 28.9% (95% CI: 23.6–34.8%) in the ST group (Figure 3B). Pneumonia incidence was also markedly reduced (RR = 0.11, 95% CI: 0.03–0.41; *p* < 0.01), occurring in 0.8% (95% CI: 0.4–1.6%) of UMF-treated patients compared with 11.5% (95% CI: 8.1–16.0%) of ST-treated patients. For otitis, the pooled analysis showed no statistically significant difference (RR = 0.43, 95% CI: 0.13–1.44; *p* > 0.05), with rates of 1.1% (95% CI: 0.6–2.0%) in the UMF group and 11.7% (95% CI: 7.3–18.2%) in the ST group. Similarly, no significant reduction was observed for sinusitis (RR = 0.59, 95% CI: 0.19–1.86; *p* > 0.05), with an incidence of 0.9% (95% CI: 0.5–1.8%) in UMF recipients versus 11.7% (95% CI: 6.8–19.4%) in controls.

In two studies, complication rates were similar between umifenovir (44.0%; 95% CI: 33.9–54.7) and oseltamivir (48.1%; 95% CI: 35.4–61.1), with no significant difference observed (RR = 0.92, 95% CI: 0.64–1.33; *p* > 0.05, Figure 3C).

### 3.5. Prophylactic Outcomes

#### 3.5.1. Incidence of ILI (Including Influenza)

**Figure 4:**
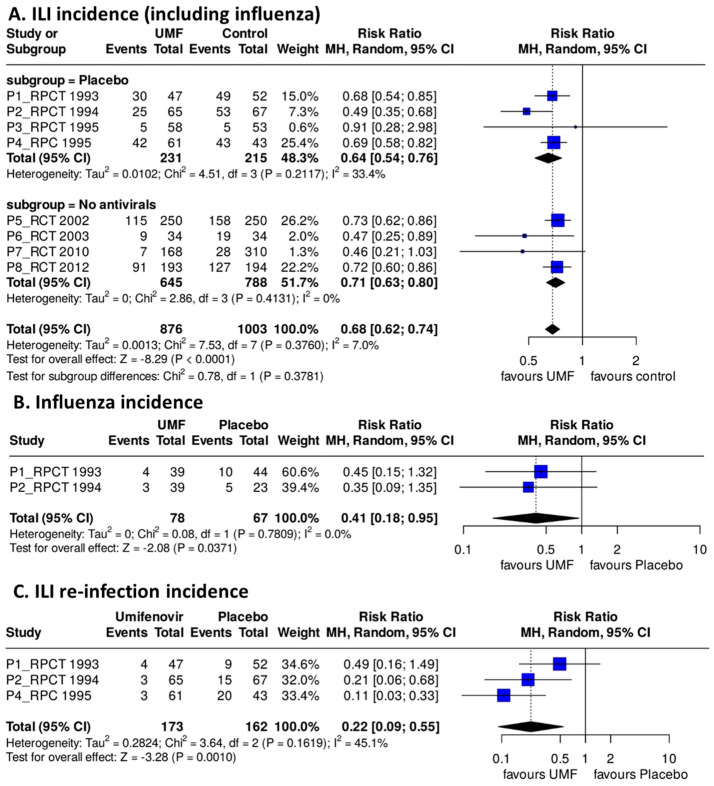
Forest plots of prophylactic efficacy of umifenovir (UMF) versus placebo or no antiviral treatment. (**A**) Incidence of ARVI and influenza; no differences in effect estimates were observed when comparing subgroups of patients receiving placebo versus those who received no antiviral therapy. (**B**) Laboratory-confirmed influenza incidence. (**C**) Risk of ARVI or influenza re-infection. Study identifiers correspond to those listed in Appendix A, Table A2.

Eight studies including 1879 participants reported (Figure 4A) ILI occurrence in 324 participants receiving UMF (37.0%; 95% CI: 33.8–40.3) and 500 participants in the control group (49.9%; 95% CI: 46.8–53.0). The pooled analysis demonstrated a reduction in risk with UMF (RR = 0.68; 95% CI: 0.62–0.74; *p* < 0.05), with no evidence of heterogeneity (I^2^ = 0%). No differences in effect estimates were observed when comparing subgroups of patients receiving the placebo versus those who received no antiviral therapy (*p* < 0.05).

#### 3.5.2. Influenza Incidence

Two placebo-controlled studies involving 145 participants assessed laboratory-confirmed influenza (Figure 4B). Influenza occurred in 7 UMF-treated participants (9.0%; 95% CI: 4.4–17.5) versus 15 placebo participants (22.4%; 95% CI: 13.9–34.1). The pooled analysis indicated reduction in risk (RR = 0.41; 95% CI: 0.18–0.95; *p* < 0.05).

#### 3.5.3. ILI Re-Infection Incidence

Three placebo-controlled studies involving 335 subjects reported re-infection incidence. A total of 10 cases were observed in the UMF group (5.8%; 95% CI: 3.2–10.3) compared with 44 cases in the placebo group (27.2%; 95% CI: 20.9–34.6) with a reduction in risk in the UMF cohort (RR = 0.22; 95% CI: 0.09–0.55; *p* < 0.05, Figure 4C).

### 3.6. Adverse Events

Across three studies that were analyzed, with a total of 162 subjects in the UMF cohort and 216 subjects in the control cohort, umifenovir demonstrated a favorable safety profile with no significant differences in the incidence of individual adverse events compared with oseltamivir or standard treatment. Rates of gastrointestinal symptoms (vomiting, abdominal pain, nausea, diarrhea), hepatic enzyme elevations, and allergic reactions were generally low, and risk ratios with 95% confidence intervals consistently crossed unity (Table 1).

**Figure 5:**
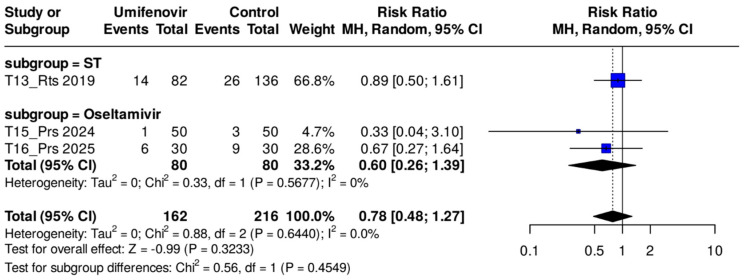
Forest plot of adverse events in patients receiving umifenovir compared with oseltamivir or standard therapy. Study identifiers correspond to those listed in Appendix A, Table A1.

There was no statistical difference between the two cohorts (RR = 0.78; 95% CI: 0.48–1.27, Figure 5). In individual trials, the incidence of adverse events with UMF ranged from 2.0% (95% CI: 0.4–10.5) to 20.0% (95% CI: 9.5–37.3), while in the control groups it ranged from 6.0% (95% CI: 2.1–16.2) to 30.0% (95% CI: 16.7–47.9). In a larger retrospective cohort, UMF showed 17.1% (95% CI: 10.5–26.6) adverse events compared to 19.1% (95% CI: 13.4–26.5) with standard therapy.

## 4. Discussion

A total of 24 clinical studies conducted between 1993 and 2024 in Russia, Ukraine, and China evaluated the efficacy and safety of umifenovir in children, enrolling ~4700 participants in therapeutic trials and nearly 2000 in prophylactic studies. Clinical studies evaluated the use of umifenovir for the prophylaxis and treatment of influenza-like illness in pediatric populations. Trials reported reductions in morbidity, symptom severity, illness duration, and viral shedding, with preventive use decreasing the risk of infection and complications several-fold.

The findings of this systematic review and meta-analysis suggest that umifenovir provides measurable clinical benefit in the treatment of ILI. Across 16 therapeutic studies, umifenovir may reduce the duration of major symptoms compared with standard therapy: fever by −1.41 days (95% CI: −1.97 to −0.84), cough by −1.15 days (95% CI: −1.50 to −0.79), intoxication by −1.37 days (95% CI: −1.83 to −0.92), and catarrhal symptoms by −1.10 days (95% CI: −1.48 to −0.72). Hospital stay was also reduced by nearly one day (MD: −0.91; 95% CI: −1.44 to −0.37). The risk of complications was reduced more than three-fold (RR: 0.34; 95% CI: 0.17–0.68), with notable decreases in bronchitis (RR: 0.27) and pneumonia (RR: 0.11).

Oseltamivir, a neuraminidase inhibitor approved for use in children including infants, is currently considered the standard antiviral therapy for influenza. Only four studies directly compared umifenovir with oseltamivir in pediatric influenza or ILI, including two randomized trials and two observational studies conducted in Russia and China. Most studies were single-center investigations with relatively small sample sizes, and three of the four studies were assessed as having a high risk of bias. Although available data suggested generally similar clinical outcomes, including complication rates (44.0% vs. 48.1%) and adverse event frequencies (8.8% vs. 15.0%; n = 80 in each group; χ^2^ = 1.49, *p* = 0.22), the limited number and methodological quality of comparative studies do not allow for firm conclusions regarding comparative efficacy between umifenovir and oseltamivir.

The prophylactic studies further demonstrated the preventive potential of umifenovir. In pooled analyses of nearly 1900 children, the incidence of influenza-like illness was reduced from 49.9% in the control group to 37.0% in those receiving umifenovir (RR: 0.68; 95% CI: 0.62–0.74). Laboratory-confirmed influenza was also reduced, occurring in 9.0% of treated children versus 22.4% of those on placebo (RR: 0.41; 95% CI: 0.18–0.95). Re-infection rates decreased nearly five-fold, from 27.2% in the placebo group to 5.8% in the umifenovir group (RR: 0.22; 95% CI: 0.09–0.55). These findings underscore the potential of umifenovir as a prophylactic measure in organized pediatric settings during seasonal epidemics. It should be noted that the evidence is limited, consisting primarily of small, older, single-center, placebo-controlled studies, which may affect the generalizability of the findings.

Despite the fact that influenza vaccination remains the primary strategy for the prevention of influenza, as recommended by the World Health Organization, routine influenza vaccination was incorporated into the Russian National Immunization Schedule only in 2006. Owing to the limited and heterogeneous reporting of vaccination history, influenza vaccination was not included as a factor in the meta-analysis. The available data did not permit subgroup analyses according to vaccination status or vaccine type. Vaccination coverage in the studied populations appears to have been low; in a pharmaco-epidemiological study, prior influenza vaccination was documented in only 10.1% of children aged 0–18 years (median age, 5 years) [26]. Consequently, influenza vaccination status was inconsistently reported across studies, and in some trials children who had received influenza vaccines or immunotropic/antiviral agents within the previous 12 months were excluded (Kladova, 2012 [27] and Uchaikin, 2002 [28]). Therefore, vaccination practices likely varied substantially across study periods, and vaccinated children, when present, may have received different seasonal influenza vaccines, predominantly trivalent formulations used during those years.

With regard to safety, umifenovir was well tolerated. Across three comparative trials (n = 378), the incidence of adverse events ranged from 2.0% to 20.0% in the umifenovir group and from 6.0% to 30.0% in the control groups, with no statistically significant differences (RR = 0.78; 95% CI: 0.48–1.27). Reported events were generally mild, most often transient gastrointestinal symptoms. Larger retrospective cohorts also confirmed a similar safety profile, with adverse event rates of 17.1% versus 19.1% in standard therapy. While some in vitro findings (e.g., hERG activity [29] and cytotoxicity in cell cultures [8]) have been reported, their clinical relevance remains uncertain and is not directly supported by in vivo data. Preclinical studies also demonstrated a favorable safety profile for umifenovir (Arbidol registration data). The compound was classified as low-toxicity (LD50 in rats > 3000 mg/kg) in studies across five mammalian species. No pathological changes were observed after 6-month administration. Comprehensive testing revealed no mutagenic, carcinogenic, allergenic, or teratogenic effects, and no adverse impact on reproductive function or offspring development. Specialized models also indicated an absence of central neurotropic activity. Furthermore, specific investigations of a pediatric powder formulation (25 mg/5 mL suspension) in immature rats showed no signs of acute or subacute (28-day) toxicity, local irritancy, or adverse effects on growth.

The evidence base is largely derived from older, non-Western studies conducted in Russia and China. The absence of umifenovir in the United States and Europe is likely related to its historical development pathway rather than definitive conclusions regarding its efficacy or safety. To our knowledge, the drug has never undergone formal evaluation by the U.S. Food and Drug Administration or the European Medicines Agency, as a complete regulatory dossier was not submitted and clinical development programs were not conducted in these regions, partly in the context of the availability of alternative antiviral therapies. In contrast, a full cycle of preclinical and clinical development was carried out in China [30]. Consequently, the evidence base is largely derived from Russia and China, where the drug is licensed and routinely used.

While our pooled analyses suggest a potential benefit of umifenovir for the treatment and prevention of influenza-like illness in children, these findings must be interpreted with caution. Consistent with this, the World Health Organization’s 2024 guidelines [4] provide only a conditional recommendation against routine use, reflecting uncertainties regarding clinical efficacy and the limited pediatric data available internationally. At the same time, umifenovir remains included in national clinical guidelines for pediatric influenza in Russia (2025) [2] and in draft recommendations for ARVI in children, as well as in several provincial influenza treatment protocols in China, highlighting its continued use in local practice. Taken together, these observations underscore that, despite apparent efficacy signals, the overall certainty of evidence remains low and findings should be contextualized within regional healthcare settings and guideline frameworks.

Broad-spectrum antiviral agents represent a promising strategy for reducing the global burden of respiratory infections. By targeting conserved viral structures or host factors essential for viral replication, such drugs could be effective against multiple respiratory viruses, including influenza viruses, respiratory syncytial virus, and coronaviruses [31]. Umifenovir is considered a broad-spectrum antiviral, and its use in children with influenza-like illness may offer potential benefits in cases of clinically diagnosed, unverified influenza, which was the focus of this systematic review and meta-analysis.

Several limitations should be acknowledged. First, the quality of the available evidence is limited by the age of many studies, with several trials conducted in the 1990s. Second, the evidence base is geographically restricted, with most studies conducted in single centers in Russia, and some trial reports were originally submitted for regulatory purposes as part of historical registration dossiers. Third, methodological limitations were common, including small sample sizes, incomplete blinding, and limited reporting of allocation concealment. Substantial heterogeneity was observed across most therapeutic outcomes (I^2^ = 84–99%), likely reflecting variability in study design, diagnostic criteria (laboratory-confirmed versus clinical influenza or broader influenza-like illness defined as fever > 37.5 °C plus respiratory symptoms), and outcome measurement. While non-randomized studies often contributed important data, particularly for secondary outcomes such as complication rates, their inclusion introduces potential biases, and differences between RCTs and non-RCTs should be interpreted cautiously. Finally, the overall certainty of evidence is low, and conclusions should be considered in the context of current international guideline recommendations, which do not provide pediatric-specific endorsements.

Although some included studies are old, the fundamental principles of conducting clinical research on non-severe influenza have remained largely unchanged over time. Study endpoints, standard therapeutic approaches (oseltamivir), and methodological procedures used in these trials are consistent with contemporary practices, supporting the continued relevance of these historical data.

Moreover, the overall quality of evidence is further limited by potential language and selection biases, as well as possible publication bias, which should be considered when interpreting the findings and their applicability to broader pediatric populations.

## 5. Conclusions

This systematic review and meta-analysis of 23 clinical studies involving nearly 6700 pediatric participants provides evidence that umifenovir (Arbidol) may offer potential benefit in the treatment and prophylaxis of influenza and influenza-like illnesses in children (low certainty). The therapeutic use of umifenovir was associated with a reduction in the duration of fever (−1.4 days), cough (−1.5 days), intoxication (−1.4 days), catarrhal symptoms (−1.1 days), and hospitalization (−0.9 days), as well as a three- to nine-fold decrease in the risk of complications such as bronchitis and pneumonia (low certainty). Prophylactic administration reduced the incidence of ILI. Across studies, umifenovir demonstrated a favorable safety profile, with adverse events comparable to the placebo, symptomatic therapy, or oseltamivir. However, the overall certainty of evidence is limited by the age, geographic restriction, and methodological quality of the included studies. While laboratory confirmation can improve diagnostic accuracy, its routine use is often restricted in pediatric care, making studies of clinically diagnosed ILI relevant to real-world practice. Further high-quality, multi-center, randomized trials are needed to confirm these findings and better inform international guideline development.

## Figures and Tables

**Table 1:** Safety outcomes of umifenovir versus comparators (ST and oseltamivir).

Study ID	Groups	System Organ Class	Preferred Term	UMF (*n*/*N*, %)	Comparator (*n*/*N*, %)
Mo, 2025 [14]	Umifenovir (*n* = 30) Oseltamivir (*n* = 30)	Gastrointestinal	Vomiting	2/30 (6.7%)	4/30 (13.3%)
		Gastrointestinal	Abdominal pain	1/30 (3.3%)	3/30 (10.0%)
		Hepatic	ALT elevation	3/30 (10.0%)	2/30 (6.7%)
Liu, 2024 [13]	Umifenovir (*n* = 50) Oseltamivir (*n* = 50)	Gastrointestinal	Nausea	0/50 (0%)	1/50 (2.0%)
		Gastrointestinal	Diarrhea	1/50 (2.0%)	1/50 (2.0%)
		Hepatic	ALT elevation	0/50 (0%)	1/50 (2.0%)
		Dermatologic	Allergy	0/50 (0%)	0/50 (0%)
Dondurey, 2019 [25]	Umifenovir (*n* = 82) ST (*n* = 134)	Dermatologic	Allergy	14/82 (17.1%)	26/134 (19.4%)

**Figure A1:**
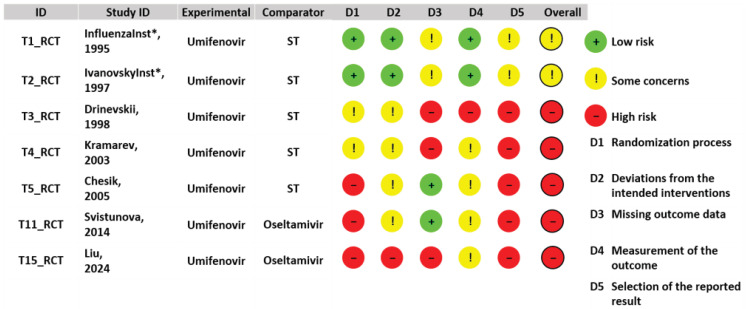
Summary risk-of-bias assessment of the included randomized studies (Table A1) using the Cochrane Risk of Bias tool (RoB 2). Study identifiers (ID) correspond to those listed in Table A1, *—full study reports and/or protocols were additionally analyzed [13,20,24,32,33,39].

**Figure A2:**
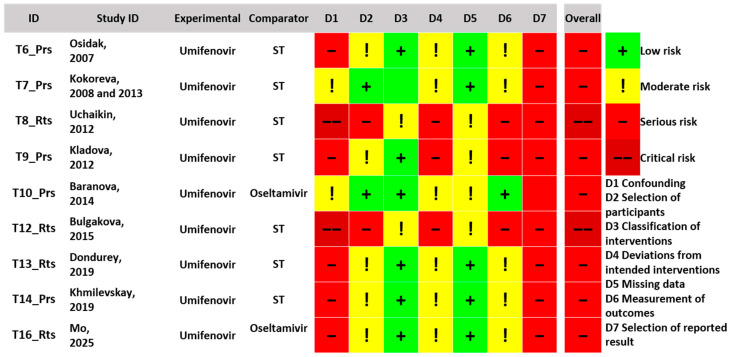
Summary risk-of-bias assessment of the included non-randomized studies (prospective and retrospective, Table A1) using the ROBINS-I tool. Study identifiers (ID) correspond to those listed in Table A1 [14,25,26,27,34,35,36,37,38,40].

**Figure A3:**
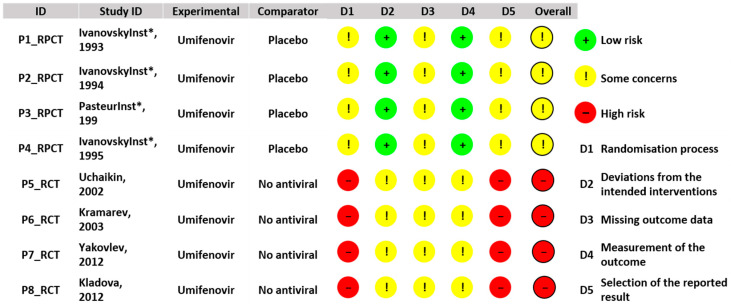
Summary risk-of-bias assessment of the included randomized studies (Table A2) using the Cochrane Risk of Bias tool (RoB 2). Study identifiers (ID) correspond to those listed in Table A1, *—full study reports and/or protocols were additionally analyzed [20,24,27,28,41].

## Data Availability

No new data were created or analyzed in this study.

## Supplementary Materials

The following supporting information can be downloaded at: https://www.mdpi.com/article/10.3390/pediatric18030077/s1, Table S1: PRISMA 2020 for Abstracts Checklist.

## Abbreviations

The following abbreviations are used in this manuscript:

AE	Adverse Event
ARVI	Acute Respiratory Viral Infection
CI	Confidence Interval
CNKI	China National Knowledge Infrastructure
CT	Clinical Trial
HR	high risk
ILI	Influenza-Like Illness
LR	low risk
MD	Mean Difference
RCT	Randomized Controlled Trial
RR	Risk Ratio
RSCI	Russian Science Citation Index
ST	standard therapy
WHO	World Health Organization

## Appendix A

### Appendix A.1

**Table A1:** Characteristics of the included studies of umifenovir in children with influenza and influenza-like illnesses (*—full study reports and/or protocols were additionally analyzed). ST—symptomatic therapy/standard care. RofB—overall risk of bias in the selected RCTs (!—some concerns; HR—high risk, Figure A1) and non-RTS assessed (SR—serious risk; CR—critical risk, Figure A2). Umifenovir was administered at age-specific single doses: 50 mg for children aged 3–6 years, 100 mg for children aged 6–12 years, and 200 mg for adolescents older than 12 years. The approved dosage regimen in Russia for the treatment of influenza and other influenza-like illnesses (ILIs) is one age-specific dose administered four times daily for five days.

ID	Study, Year (Dosage Form)	N	Study Design	Main Diagnosis	Age	Outpatient/Hospitalized	Inclusion Criteria	Comparison	Dosage Regime	Setting	RofB
T1_RCT	InfluenzaInst *, 1995 [20] (tablet, 50 mg)	158	Randomized controlled trial	ILI (Influenza A—40% and B—3%)	1—14	Hospitalized	Children with ILI (moderate severity)	ST	4 SD per day for 5 days	Single-center (Russia)	!
T2_RCT	IvanovskyInst *, 1997 [20] (tablet, 50 mg)	120	Randomized controlled trial	ILI with stenosing laryngotracheitis	3–11	Hospitalized	Within 2 days of ILI onset, no prior antiviral therapy	ST	4 SD per day for 5 days	Single-center (Russia)	!
T3_RCT	Drinevskii, 1998 [32] (tablet, 50 mg)	158	Randomized controlled trial	Influenza A and ILI	1–14	Both	Children with ILI symptoms	ST	4 SD per day for 5 days	Multi-center	HR
T4_RCT	Kramarev, 2003 [24] (tablet, 100 mg)	68	Randomized controlled trial	ILI	6–15	Both	Children with ILI symptoms	ST	4 SD per day for 5 days	Single-center (Ukraine)	HR
T5_RCT	Chesik, 2005 [33]	90	Randomized controlled trial	ILI with obstructive laryngitis	2–6	Hospitalized	Within 3 days of ILI onset	ST	4 SD per day for 5 days	Single-center (Russia)	HR
T6_Prs	Osidak, 2007 [34]	255	Prospective	ILI	2–17	Hospitalized	n.d.	ST	4 SD per day for 5 days	Single-center (Russia)	SR
T7_Prs	Kokoreva, 2008 [35], 2013 [36]	94	Prospective	ILI	2–14	Hospitalized	Children with ILI symptoms	ST	4 SD per day for 5 days	Single-center (Russia)	SR
T8_Rts	Uchaikin, 2012 [37]	950	Retrospective	Influenza and ILI	0–17	Hospitalized	Clinical diagnosis of influenza/ARVI	ST	4 SD per day for 5 days	Multi-center (37 hospitals across Russia)	CR
T9_Prs	Kladova, 2012 [27]	42	Prospective	ILI	7–12	Outpatient	Children who fell ill within 3 months after prophylactic	ST	4 SD per day for 5 days	Single-center (Russia)	SR
T10_Prs	Baranova, 2014 [38]	300	Prospective	Influenza	3–17	Hospitalized	Within 2 days of onset	Oseltamivir	4 SD per day for 5 days	Single-center (Russia)	SR
T11_RCT	Svistunova, 2014 [39]	104	Randomized controlled trial	Influenza A or B (lab-confirmed)	3–17 (10.9)	Hospitalized	PCR-confirmed influenza, moderate severity	Oseltamivir	4 SD per day for 5 days	Single-center (Russia)	HR
T12_Rts	Bulgakova, 2015 [26]	2044	Retrospective	Influenza A and ILI	2–17	Hospitalized	Fever (>38 °C) and at least one of the following symptoms: cough, sore throat, rhinorrhea	ST	4 SD per day for 5 days	Multi-center (15 sites)	CR
T13_Rts	Dondurey, 2019 [25]	216	Retrospective	Influenza A and ILI	2–17	Hospitalized	Within 3 days of onset	ST	4 SD per day for 5 days	Single-center (Russia)	SR
T14_Prs	Khmilevskaya, 2019 [40]	80	Prospective	ILI	3–12	Hospitalized	Within 2 days of onset	ST	4 SD per day for 5 days	Single-center (Russia)	SR
T15_RCT	Liu, 2024 [13]	100	Randomized controlled trial	Influenza	2–6 (3)	Outpatient	Non-severe ILI, >38 °C within 2 days onset	Oseltamivir	3 SD per day for 5 days	Single-center (China)	HR
T16_Rts	Mo, 2025 [14]	60	Retrospective	Influenza (lab-confirmed)	2–13 (5.8)	Hospitalized	Acute ILI, fever > 38, cough (moderate severity)	Oseltamivir	3 SD per day for 5 days	Single-center (China)	SR

**Table A2:** Characteristics of the included studies assessing the prophylactic efficacy of umifenovir in children against influenza and influenza-like illnesses (*—full study reports and/or protocols were additionally analyzed). All studies were single-center and conducted in Russia, except for Kramarev, 2003 (Ukraine) [24]. RofB, overall risk of bias in the selected studies (!—some concerns, HR—high risk, Figure A3). Umifenovir was administered according to age-specific single-dose regimens: 50 mg for children aged 3–6 years, 100 mg for children aged 6–12 years, and 200 mg for adolescents older than 12 years.

ID	StudyID, Year (Dosage Form)	N	Endpoints	Inclusion Criteria	Comparison	Age	Outpatient/Hospitalized	Study Design	Dosage Regime	Duration	RofB
P1_RPCT	IvanovskyInst *, 1993 [20] (tablet, 50 mg)	99	ILI and laboratory-confirmed influenza A or B	Children during influenza epidemic seasons	Placebo	6–15	Outpatient	Randomized placebo-controlled	SD two times per week	23 days	!
P2_RPCT	IvanovskyInst *, 1994 [20] (tablet, 50 mg)	132	ILI and laboratory-confirmed influenza A or B	Children during influenza epidemic seasons	Placebo	6–15	Outpatient	Randomized placebo-controlled	SD three times per week	5 weeks	!
P3_RPCT	PasteurInst *, 1995 [20] (tablet, 50 mg)	155	Influenza and ILI (parainfluenza)	Children during influenza epidemic seasons	Placebo	3–7	Outpatient	Randomized, double-blind placebo-controlled	50 mg three times per week	3 weeks	!
P4_RPCT	IvanovskyInst *, 1995 [20] (tablet, 50 mg)	104	ILI and laboratory-confirmed influenza A or B	Children during influenza epidemic seasons	Placebo	6–15	Outpatient	Randomized placebo-controlled	SD three times per week	4 weeks	!
P5_RCT	Uchaikin, 2002 [28] (tablet, 100 mg)	500	ILI (within 3 months)	Children randomized by stratified method by sex, age, infection history, diagnosis in past year, and prophylactic regimen	No antiviral (preventive hygiene measures)	6–17	Outpatient	Randomized controlled trial	SD two times per week	3 weeks	HR
P6_RCT	Kramarev, 2003 [24] (tablet, 100 mg)	156	ILI	Children randomized by stratified method	No antiviral	6–15	Outpatient	Randomized controlled trial	100 mg once every 3 days	4 weeks	HR
P7_RCT	Yakovlev, 2012 [41] (capsules, 100 mg)	478	Influenza or ILI	Unvaccinated children against influenza	No antiviral	7–10	Outpatient	Pseudo-randomized controlled cohort study	100 mg two times per week	3 weeks	HR
P8_RCT	Kladova, 2012 [27] (capsules, 100 mg)	362	ILI (within 3 months)	Frequently ill children and with chronic diseases	No antiviral	7–12	Outpatient	Randomized controlled trial	100 mg two times per week	4 weeks	HR
